# Supplementary material for: Regulatory T cells promote cancer immune-escape through integrin αvβ8-mediated TGF-β activation
Source: Nat Commun. 2021 Oct 28;12:6228. doi: 10.1038/s41467-021-26352-2 (PMC8553942; doi:10.1038/s41467-021-26352-2)
Supplement: Supplementary file 1 — Supplementary Information [file 41467_2021_26352_MOESM1_ESM.pdf]

Regulatory T Cells Promote Cancer Immune-escape Through Integrin αvβ8-mediated TGF-β Activation

Supplementary Fig. 1

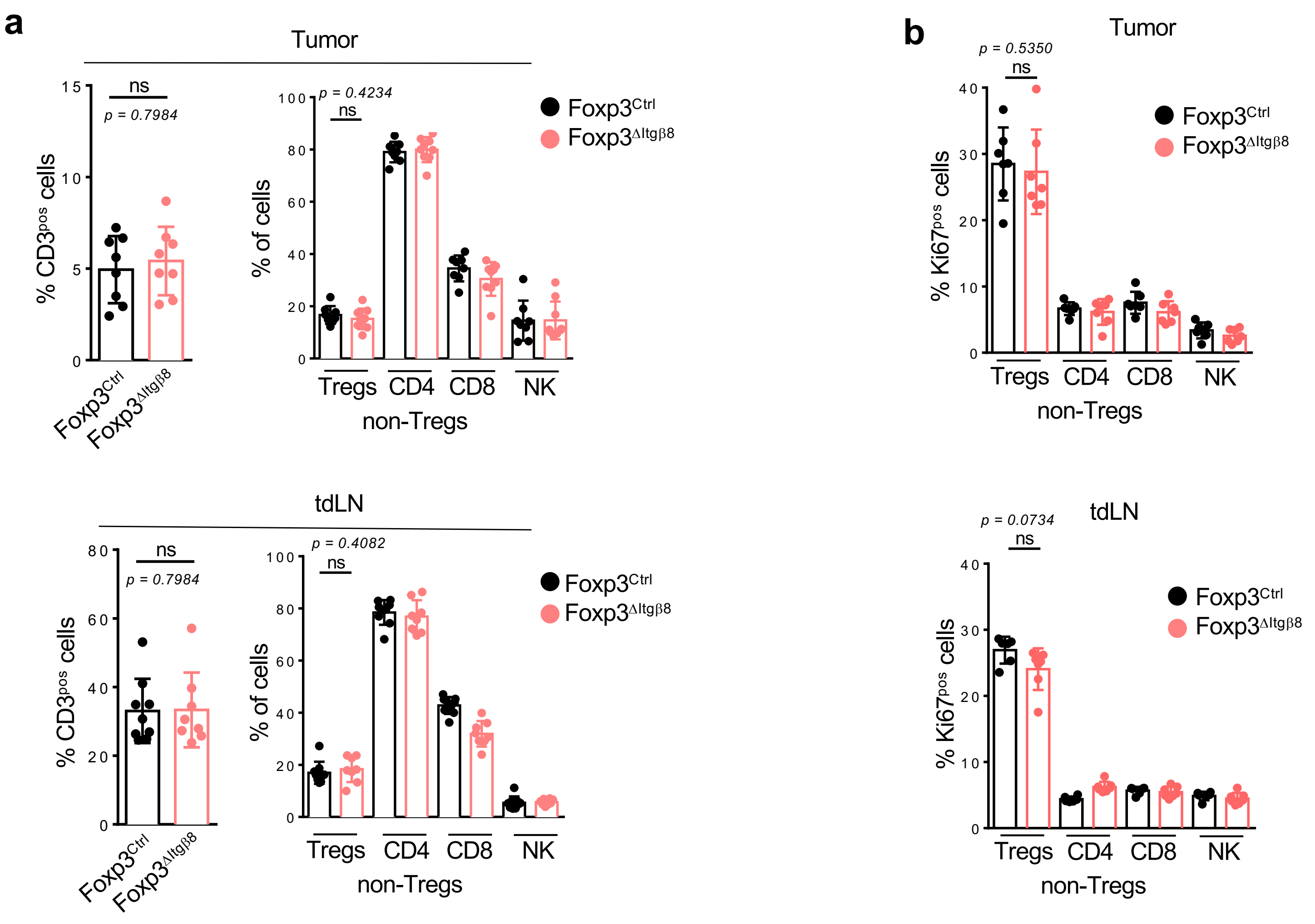

Supplementary figure 1: Itgβ8 Tregs do not impact homeostasis of tumor infiltrating immune cells

Foxp3Ctrl and Foxp3ΔItgβ8 mice were injected i.d. with B16 cells in the back. Both Tumor and tumor-draining lymph nodes (tdLN) were analyzed by flow cytometry day 15 after tumor implantation. **a**) Graphs illustrates the percentage of T cells among the hematopoietic cells of different effectors cells and Tregs. **b**) Graphs demonstrate the percentage of cells Ki67pos cells among Tregs and different effector immune cell populations. Data are representative of 2 experiments with 7-8 mice per groups. Means are shown ± SD. was determined by two-tailed Mann Whitney test. ns: statistically not significant. Source data are provided as a Source Data file.

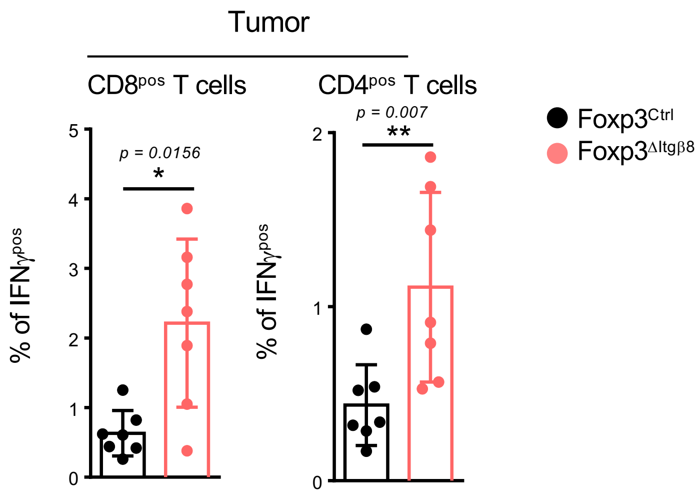

Supplementary figure 2: Itgβ8pos Tregs impair IFN–γ production of T cells in the tumor

T cells infiltrating the tumor (B16) were analyzed for their ability to produce IFN-γ 15 days after implantation. Graph demonstrates the percentage of IFN-γ positive cells among either CD8pos T cells or CD4pos T cells from different animals (n=7) from 2 independent experiments. $^{**}p < 0.01$, $^{*}\ p < 0.05$; unpaired two tailed Student's t, mean ± SD. $p = 0.0156$ (*) / $p = 0.0070$ (**). Source data are provided as a Source Data file.

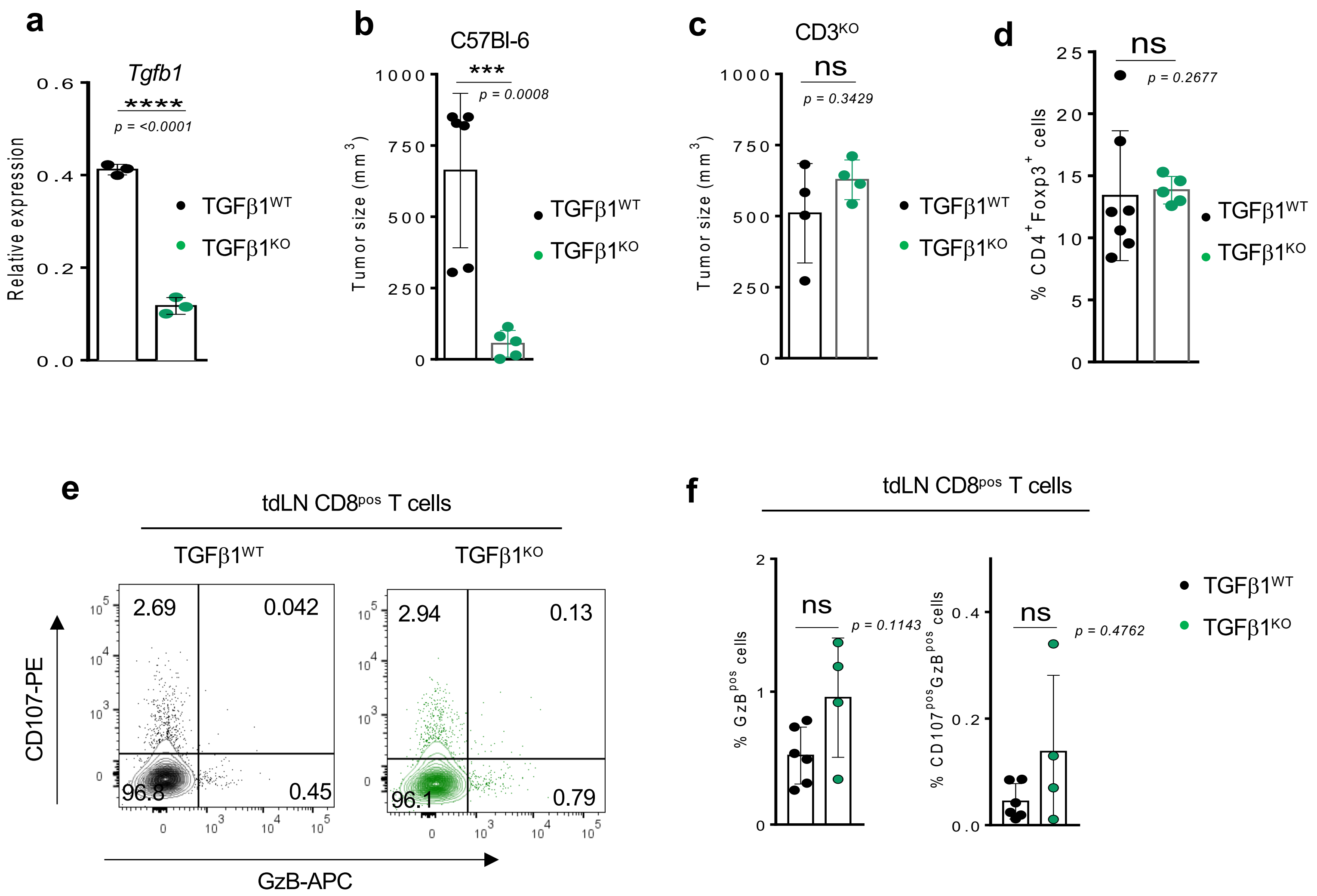

Supplementary figure 3: TGFβ1 produced by cancer cells is essential to control T cell anti-tumor response

a) Graph demonstrates the expression levels *Tgfb1* in B16 *shTgfb1* (TGFβ1^{KO}) B16 cells as well as in their TGFβ1^{CTRL} control cell line after RTqPCR analysis and normalization with *gadph* expression. TGFβ1^{KO} B16 cells and TGFβ1^{CTRL} B16 cells were injected i.d either C57Bl/6 mice (**b, d-f**) or in CD3^{KO} mice (**c**). B and C illustrate the final volume of the tumors 15 days later. **d**) Graph illustrates the percentages of Tregs among CD4 T cells within the tumors obtained after flow cytometry analysis. (**e-f**) CD8^{pos} T cells from the tumor draining lymph node (tdLN) were analyzed by flow cytometry Representative counter plots illustrating the cytotoxic functions are shown (E) as well as graphs demonstrating the percentage of CD8^{pos} T cells expressing either granzyme B (GzB) and CD107 (F), data are representative for 4-6 tumors per group. For bar graphs means are shown ± SD. ** $p < 0.001$; **** $p < 0.0001$ unpaired two tailed Student's t test for A-C. Mann Whitney test was used for E-F. ns: statistically not significant. Sup 3A *p*= <0.0001, sup 3B *p*= 0.0008. Source data are provided as a Source Data file.

Supplementary Fig. 4

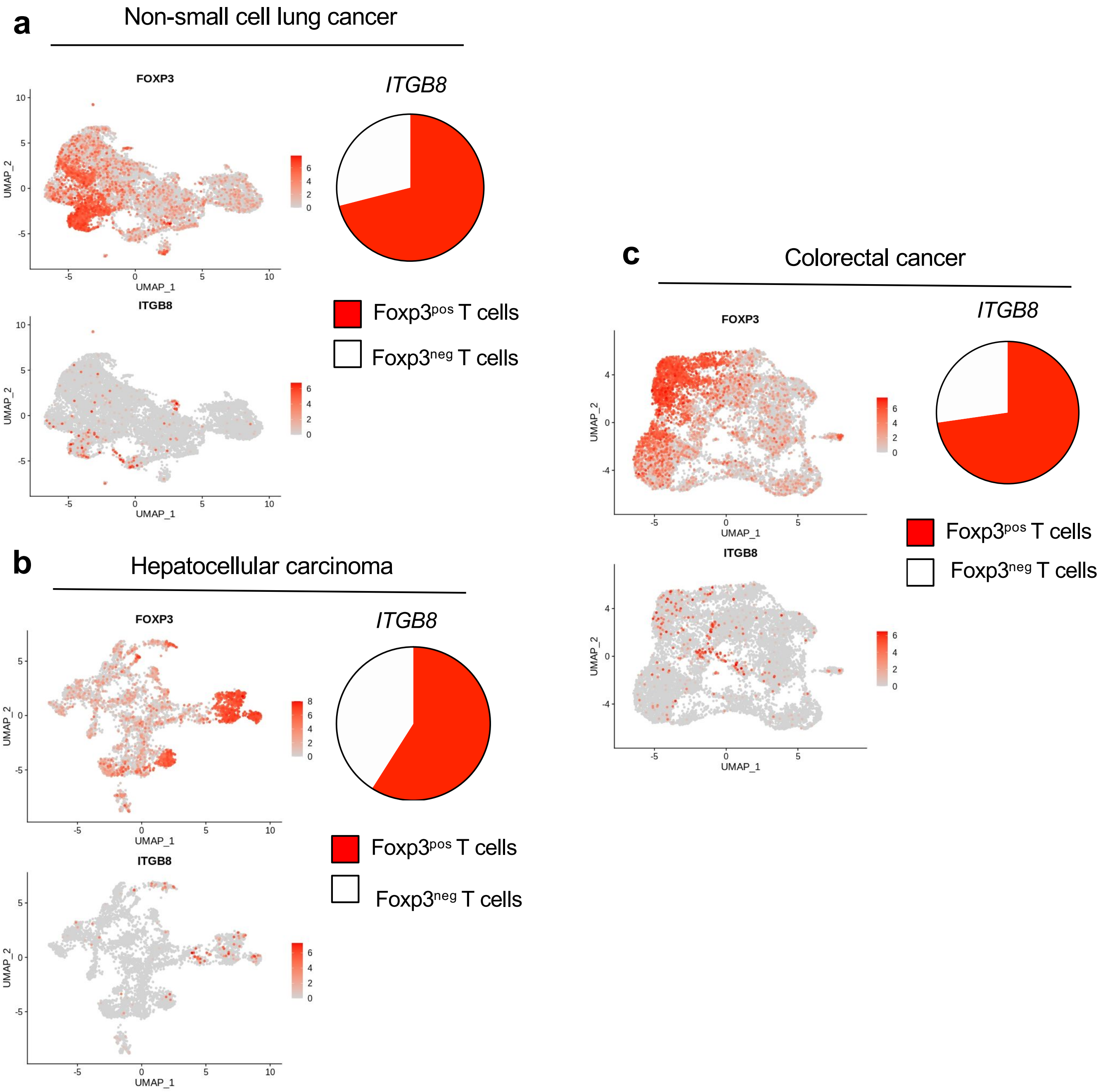

Supplementary figure 4: *ITGB8* is expressed on tumor infiltrating Tregs in patients
Transcriptomic analysis from single cell RNAseq data obtained T cell infiltrating non-small cell lung cancer (**a**), hepatocellular carcinoma (**b**) and colorectal cancer (**c**). DimPlots and pie-charts show the proportion of *ITGB8*-expressing cells among *FOXP3* negative and *FOXP3* positive T cells for each of the datasets of the different tumors. Source data are provided as a Source Data file.

Supplementary Fig. 5

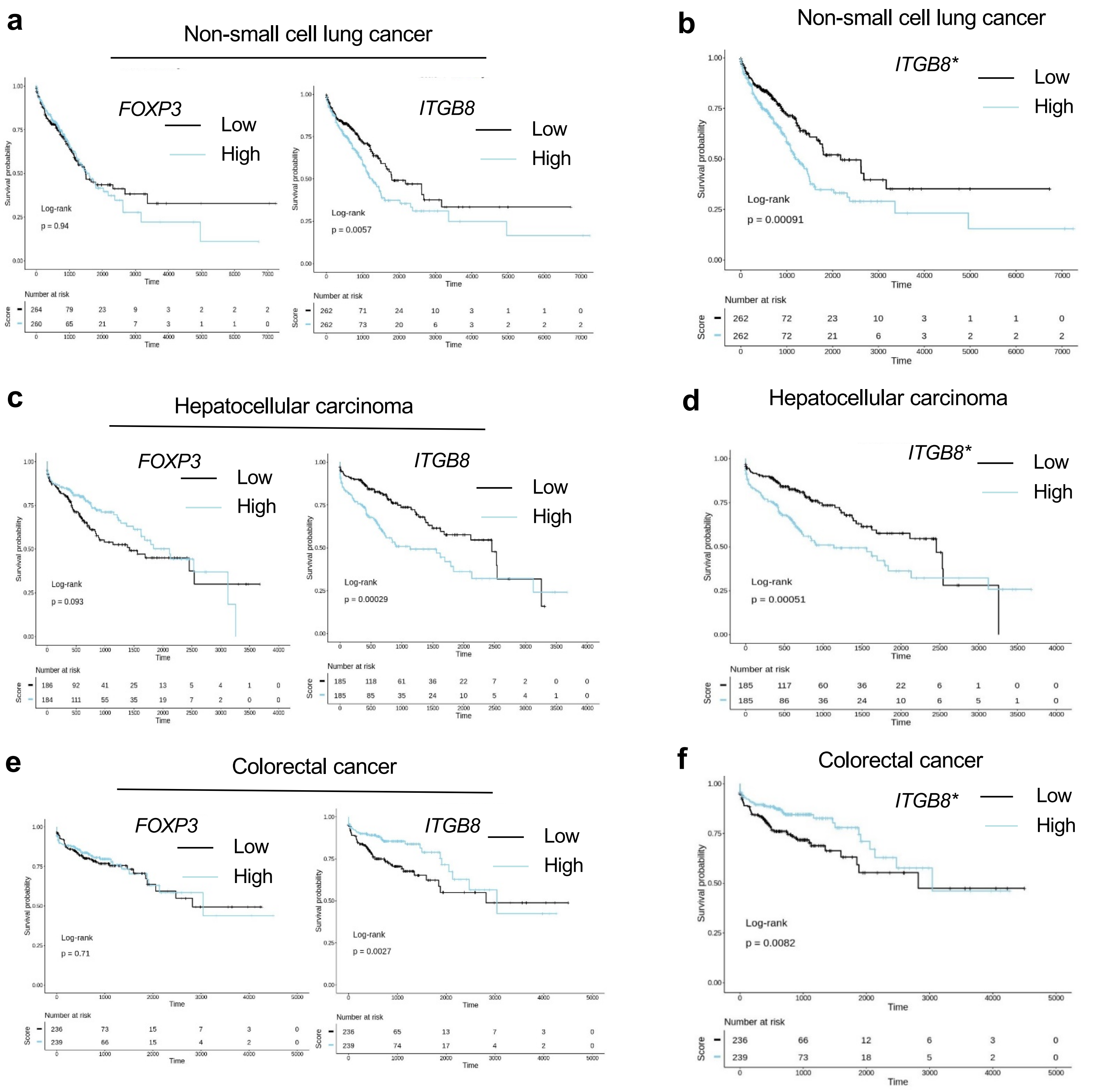

Supplementary figure 5: High Itgβ8 score on TME T cell correlates with patient worse prognostic in several tumor types

Transcriptome signatures of Itgβ8^{pos} Treg cells deprived of Treg activation signature or not from non-small cell lung cancer (**a-b**), hepatocellular carcinoma (**c-d**) and colorectal cancer (**e-f**) were extracted from single cell RNAseq data, (as described in Methods). The extracted signatures were used for testing their association with overall survival on The Cancer Genome Atlas (TCGA) RNAseq data from their respective tumor type. Tumor samples were classified into Low and High, based on the expression of *FOXP3*, *ITGB8* score and ITG8 deprived of activation (*ITGB8*)*. Graph illustrate the overall survival of cancer patients stratified by these scores in tumor infiltrating T cells with time expressed in days. n = 524/290/572 patients for non-small cell lung cancer, hepatocellular carcinoma and colorectal cancer respectively. log rank value are mentioned on the graphs based Mantel-Cox test.

Supplementary Fig. 6

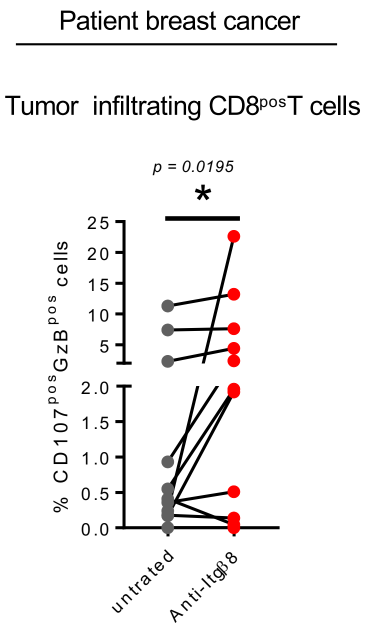

Supplementary figure 6: anti-Itgβ8 antibody treatment increases cytotoxic functions CD8 T cells infiltrating breast cancer patients

Fresh serial sections from a same breast cancer for each patient were maintained in culture conditions in the presence or not of neutralizing anti-Itgβ8 antibody. 48 hours later $CD8^{pos}$ T cells infiltrating the tumors were analyzed by flow cytometry. Graph illustrates the percentage of $CD8^{pos}$ T cells positive for granzyme B (GzB) and CD107, confirming the degranulation, in response to treatment. Each grey dot illustrates the value for tumor $CD8^{pos}$ T cells in the absence of treatment and the linked red dot that of $CD8^{pos}$ T cells from the same tumor after anti-Itgβ8 antibody treatment. n=11 different patients, *$p < 0.05$ was determined by two-tailed Wilcoxon test. p= 0.0195. Source data are provided as a Source Data file.

Gating strategy used for Figure 1a

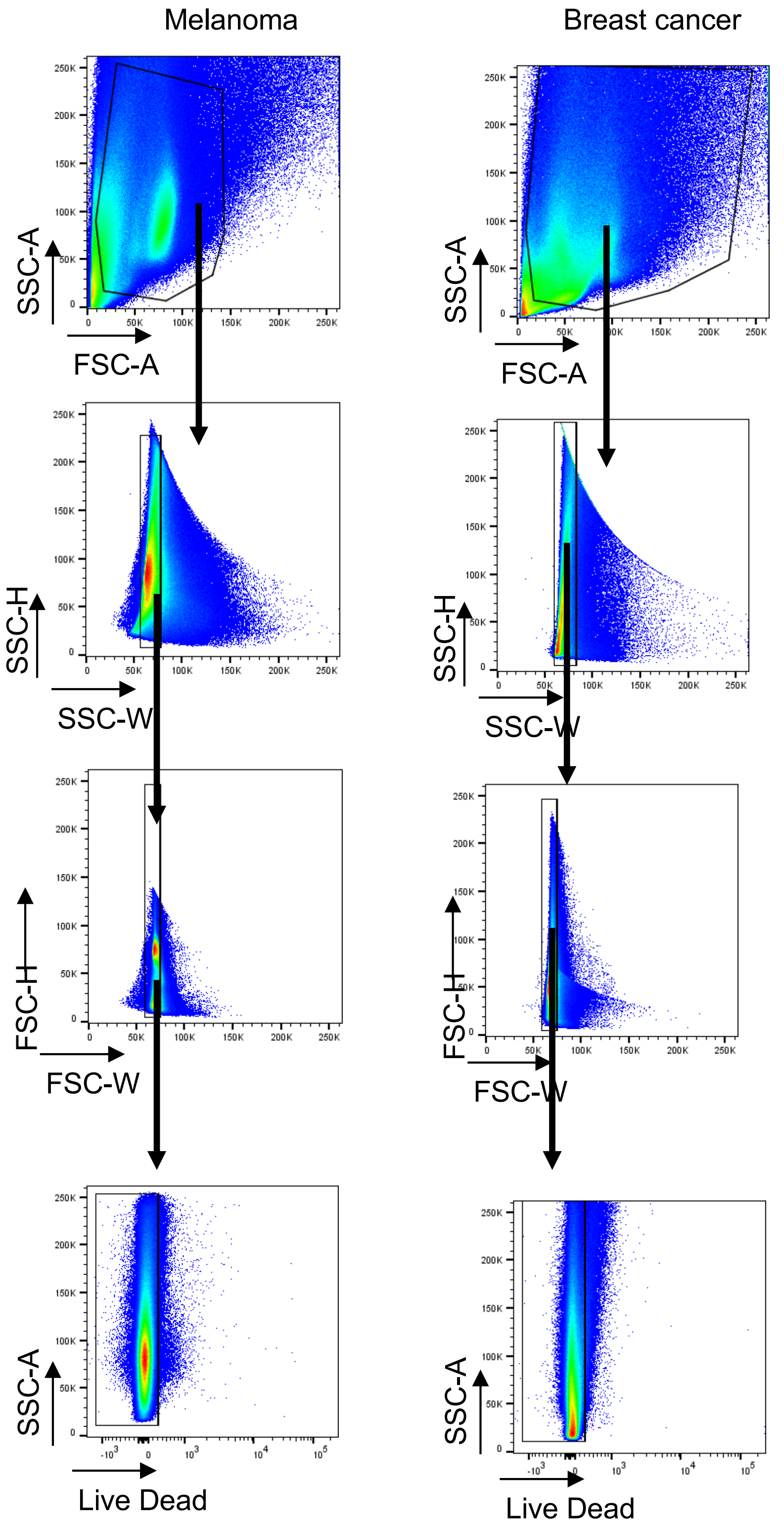

Gating strategy used for Figure 1g

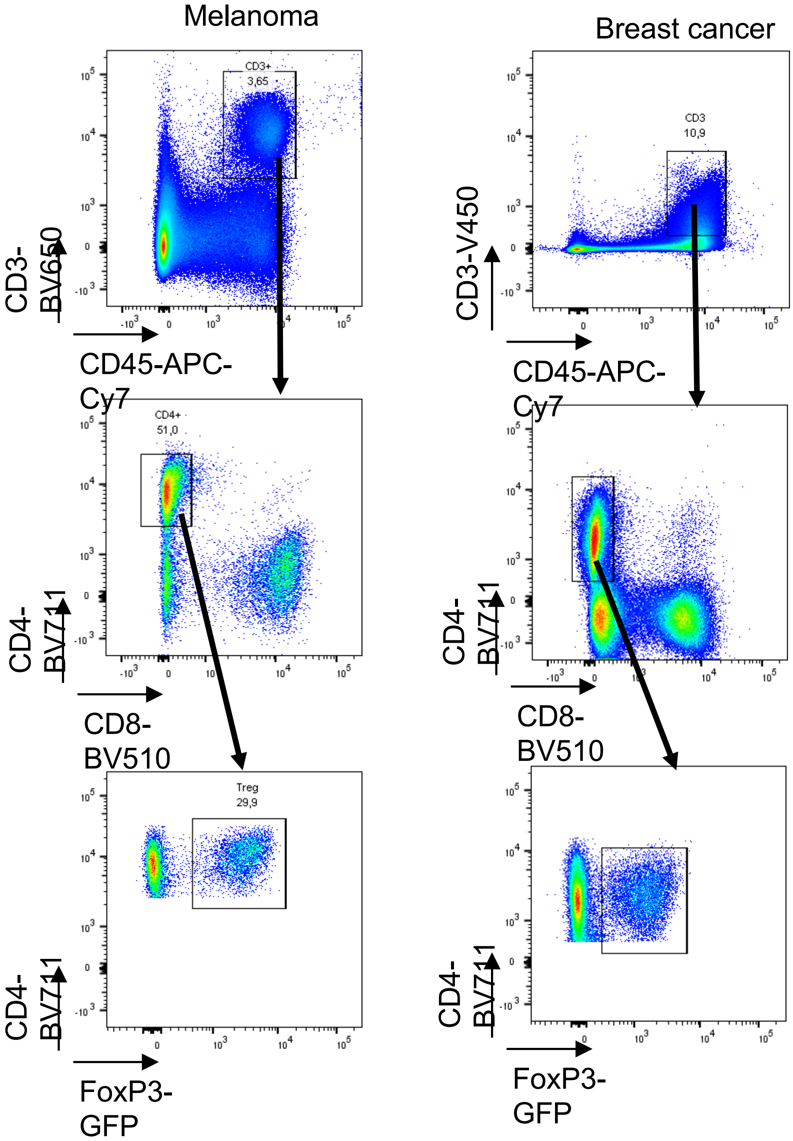

Gating strategy used for the analysis of tumor T cells in fig 3c-f Fig 4 a-e and Figure 5e

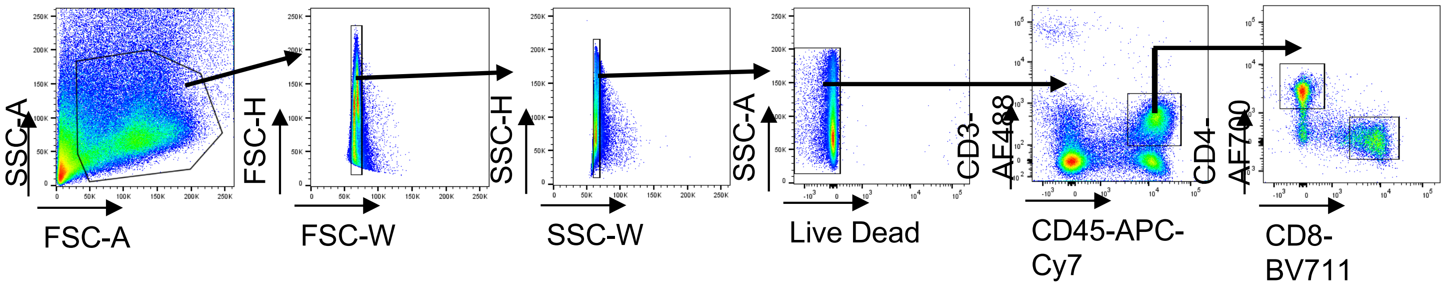

Supplementary Fig.10

Gating strategy used for the analysis of tumor draining lymph node T cells in Fig 3f and supplementary Fig 3e

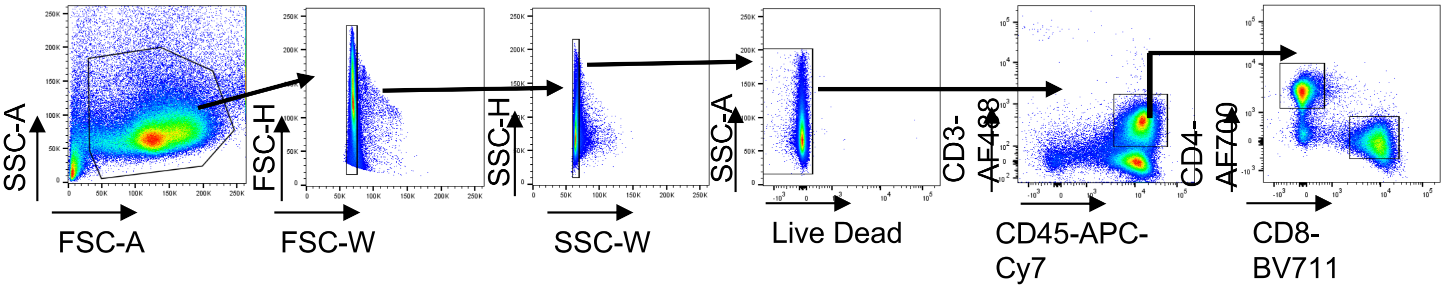

a

Gating strategy used for supplementary figure 1a (tumor)

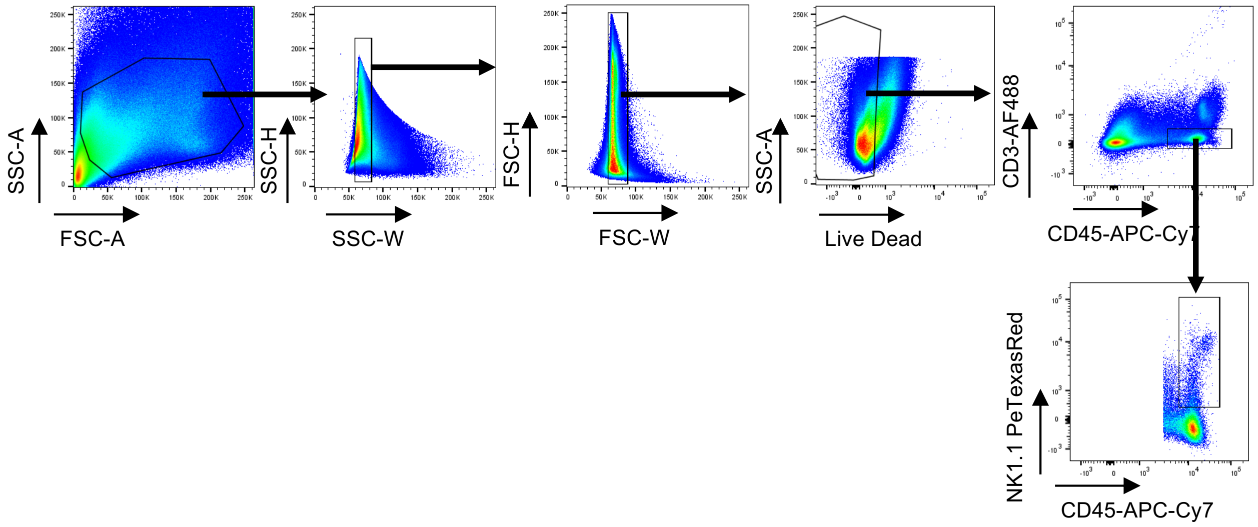

b

Gating strategy used for suplementary figure 1a (tdLN)

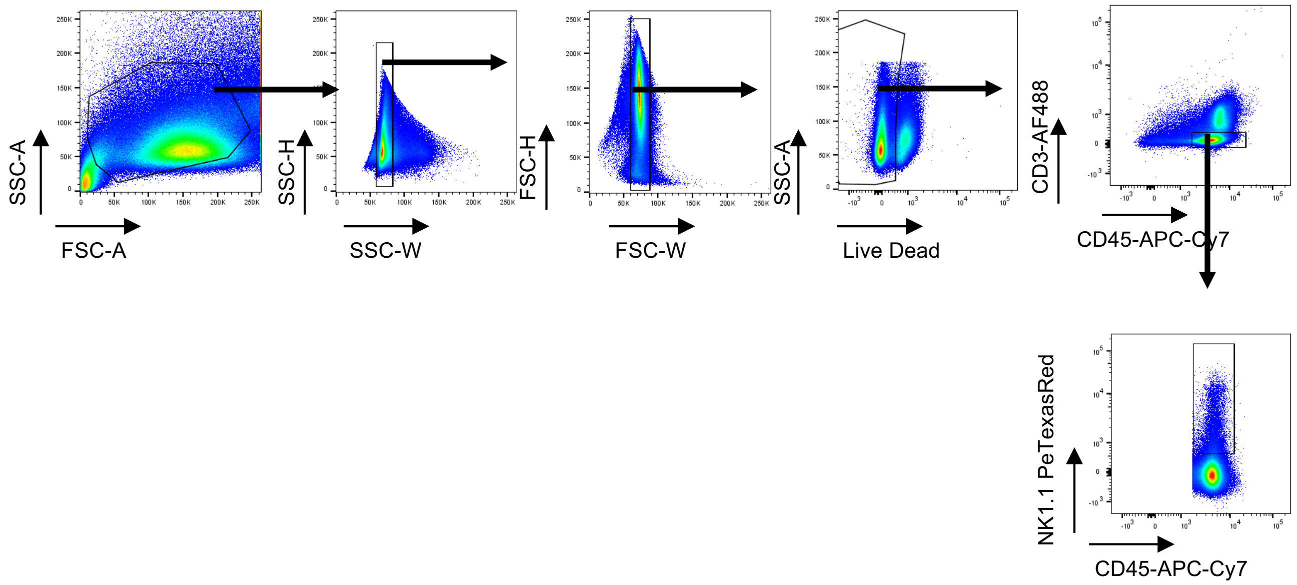

Gating strategy used for supplementary Figure 2

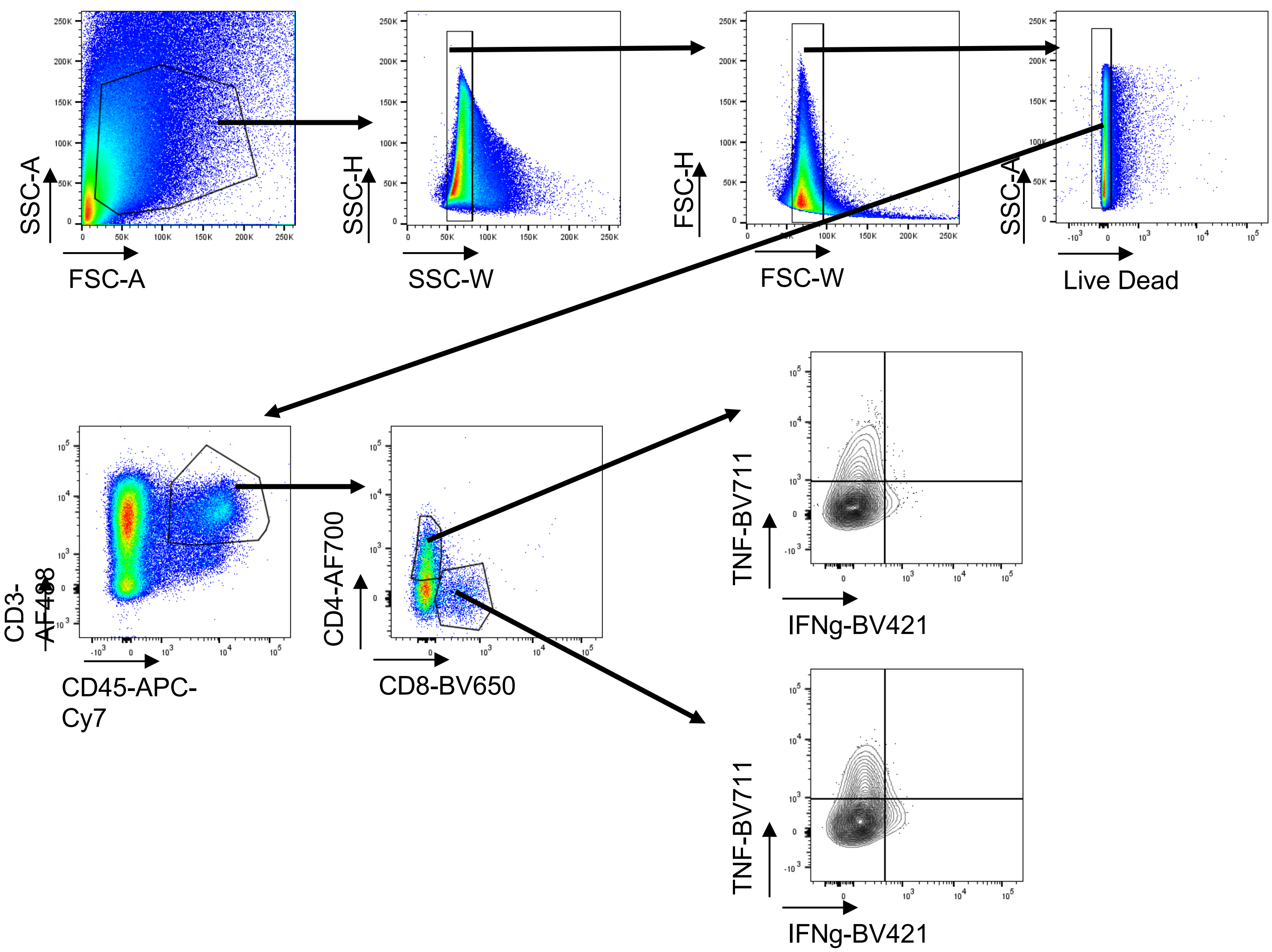

Supplementary Table 1

Primer sequences used for Q-PCR

Primers	Sequence 5’ → 3’
mTGF-β1 (forward)	ATCCTGTCCAAACTAAGGCTCG
mTGF-β1 (reverse)	ACCTCTTTAGCATAGTAGTCCGC
GAPDH (forward)	AGGTCGGTGTAACGGATTTG
GAPDH (reverse)	TGTAGACCATGTAGTTGAGGTCA
