# Supplementary material for: Regulatory T cells promote cancer immune-escape through integrin αvβ8-mediated TGF-β activation
Source: Nat Commun. 2021 Oct 28;12:6228. doi: 10.1038/s41467-021-26352-2 (PMC8553942; doi:10.1038/s41467-021-26352-2)
Supplement: Supplementary file 3 — Reporting Summary [file 41467_2021_26352_MOESM3_ESM.pdf]

nature research

Corresponding author(s): Julien C. Marie

Last updated by author(s): Aug 10, 2021

Reporting Summary

Nature Research wishes to improve the reproducibility of the work that we publish. This form provides structure for consistency and transparency in reporting. For further information on Nature Research policies, see our Editorial Policies and the Editorial Policy Checklist.

Statistics

For all statistical analyses, confirm that the following items are present in the figure legend, table legend, main text, or Methods section.

n/a	Confirmed	
☐	☒	The exact sample size (*n*) for each experimental group/condition, given as a discrete number and unit of measurement
☐	☒	A statement on whether measurements were taken from distinct samples or whether the same sample was measured repeatedly
☐	☒	The statistical test(s) used AND whether they are one- or two-sided<br>*Only common tests should be described solely by name; describe more complex techniques in the Methods section.*
☒	☐	A description of all covariates tested
☒	☐	A description of any assumptions or corrections, such as tests of normality and adjustment for multiple comparisons
☐	☒	A full description of the statistical parameters including central tendency (e.g. means) or other basic estimates (e.g. regression coefficient) AND variation (e.g. standard deviation) or associated estimates of uncertainty (e.g. confidence intervals)
☐	☒	For null hypothesis testing, the test statistic (e.g. *F*, *t*, *r*) with confidence intervals, effect sizes, degrees of freedom and *P* value noted<br>*Give P values as exact values whenever suitable.*
☒	☐	For Bayesian analysis, information on the choice of priors and Markov chain Monte Carlo settings
☒	☐	For hierarchical and complex designs, identification of the appropriate level for tests and full reporting of outcomes
☒	☐	Estimates of effect sizes (e.g. Cohen's *d*, Pearson's *r*), indicating how they were calculated

Our web collection on statistics for biologists contains articles on many of the points above.

Software and code

Policy information about availability of computer code

Data collection	description is provided in the methods, All code used in this study was based on the following R packages: Seurat v.3.1.0 (doi: https://doi.org/10.1016/j.cell.2019.05.031), singscore v1.10.0 (doi: 10.1186/s12859-018-2435-4), TCGAbiolinks v.2.9.4 (doi: 10.1093/nar/gkv1507), RTCGA.clinical v.20151101.8.0, edgeR v.3.31.1 (doi: 10.1093/bioinformatics/btp616), survival v.2.44-1.1 (https://github.com/therneau/survival), and survminer v.0.4.5 (https://rpkgs.datanovia.com/survminer/index.html). No additional code/packages were developed in the course of this study.
Data analysis	description is provided in the methods

For manuscripts utilizing custom algorithms or software that are central to the research but not yet described in published literature, software must be made available to editors and reviewers. We strongly encourage code deposition in a community repository (e.g. GitHub). See the Nature Research guidelines for submitting code & software for further information.

Data

Policy information about availability of data

All manuscripts must include a data availability statement. This statement should provide the following information, where applicable:

- Accession codes, unique identifiers, or web links for publicly available datasets
- A list of figures that have associated raw data
- A description of any restrictions on data availability

Gating strategies have been provided in " the online supplementary material section", raw data are available in "the data availability "section

Field-specific reporting

Please select the one below that is the best fit for your research. If you are not sure, read the appropriate sections before making your selection.

☒ Life sciences ☐ Behavioural & social sciences ☐ Ecological, evolutionary & environmental sciences

For a reference copy of the document with all sections, see nature.com/documents/nr-reporting-summary-flat.pdf

Life sciences study design

All studies must disclose on these points even when the disclosure is negative.

Sample size	No formal power calculation were performed. The size of each groups was chosen according both statistical robustance for the type of experiment and ethic committee agreements. Data wereroutinely collected across independant replicates for each assay. Number of samples /mice and the number of independent experiments are indicated in the figure legends
Data exclusions	No data were excluded from the analysis
Replication	All experiments were repeated with complet independent set of mice. All attemps at replication were succesful. For human sample analysis, each patient tumor being its own control (treated and not treated), sample analysis was made independently but with same antibodies lot and cytometrer calibration.
Randomization	No formal randomization was done as we compared mice with diffrent genotypes. For patients beside the gender selection (for breast cancer) no covarient (Age, ethnic, gender...) were taken in consideration. Only patient with primary tumors, and previous anti-cnacer treatments were included
Blinding	Investigators were blinded to the mouse genotypes, during tumor measurement, and histology. For all Facs staining process and acquisition (mouse and human samples) investigators were blinded as well. For data based analysis the blinding was not performed given that the analysis was not made on treated and not treated samples.

Reporting for specific materials, systems and methods

We require information from authors about some types of materials, experimental systems and methods used in many studies. Here, indicate whether each material, system or method listed is relevant to your study. If you are not sure if a list item applies to your research, read the appropriate section before selecting a response.

Materials & experimental systems

n/a	Involved in the study
☐	☒ Antibodies
☐	☒ Eukaryotic cell lines
☒	☐ Palaeontology and archaeology
☐	☒ Animals and other organisms
☐	☒ Human research participants
☒	☐ Clinical data
☒	☐ Dual use research of concern

Methods

n/a	Involved in the study
☒	☐ ChIP-seq
☐	☒ Flow cytometry
☒	☐ MRI-based neuroimaging

Antibodies

Antibodies used	anti mouse antibodies : anti- CD3e (145-2C11; BD biosciences), CD4 (RM4-5; Biolegend), CD8a (53.6.7; BD biosciences), CD45 (30-F11; BD biosciences), CD107a (eBio1D4B, eBiosciences). Granzyme B (GRB05 Invitrogen), Ki67 SolA15; ThermoFisher) IFN-g (XMG1.2 BD bioscience). anti-mouse GP100 (ab137078 Abcam) and or LAP-PE (TW7-16B4, Biolegend).<br>anti human anitibodies CD3 (UCHT1; BD biosciences), CD45 (HI30; BD biosciences), CD4 (RPA-T4; LifeTechnologies), CD8 (SK1; BD biosciences), CD107a (H4A3; BD biosciences) and Granzyme B (GRB05 Invitrogen). Neutralizing Itgb8 antibody ADWA-16 was provided by by prof Sheppard UCSf, CA USA)<br>p-SMAD2/3 (D27F4, Cell Signaling) was detected with goat anti-rabbit A488 (LifeTechnologies, A11034).<br>anti-CD8a (BioXCell, clone Lyt3.2; BE0223) was used for CD8 t cell depletion
Validation	All the antibodies , but neuralizing anti-itgb8 antibody are commercial and their validation for the technic we used is exposed on provider websites.<br>Validation of neuralizing anti-itgb8 antibody was cinfirmed in ref 38<br>Validation of p-SMAD2/3 with and without stimulation of the cells with Tgf-b was made (data avaiblity section).<br>Validation of anti-CD8b (BioXCell, clone Lyt3.2; BE0223) is illsitrating by analyzing the presence of CD8a pos T cells

Eukaryotic cell lines

Policy information about cell lines

Cell line source(s)	B16-F10 (melanoma), E0711(breast cancer), MLEC (bioactive TGF-b reporter cell line)
Authentication	authentication numbers for B16-F10 : CRL-6475 (ATCC), E0771 : CVCL_GR23 (ATCC), MLEC were provided by Prof Rifkin (NYU, NY, USA) who generated them (please see ref 38)
Mycoplasma contamination	all cells lines were tested for mycoplasma and were negative the methods of detection will be added
Commonly misidentified lines (See ICLAC register)	No commonly misidentified cell lines were used

Animals and other organisms

Policy information about studies involving animals; ARRIVE guidelines recommended for reporting animal research

Laboratory animals	mus musculus, female and male, adults (2-6 months of age), on C57BL6 background
Wild animals	No wild animals were used in this study
Field-collected samples	No filed-collected samples were used in this study
Ethics oversight	Animal Ethic Evaluation Committee (CECCAPP) and French ministry of Rese arch validate mouse protocoles (#9239 and #19584).

Note that full information on the approval of the study protocol must also be provided in the manuscript.

Human research participants

Policy information about studies involving human research participants

Population characteristics	Patient samples were obtained according the frecnh law and aproved protocoles . Protocol number is mentionned in them methods
Recruitment	No restriction of age (but over 18 y.o) gender (except for breast cancer), body weigth, size, ethnical ...was considered. Patients with only primary tumor and that never received anti-cancer treatments prior surgery were selected
Ethics oversight	Protocol number from the French ministry of research AC-2013-1871 and AC -2019-3426

Note that full information on the approval of the study protocol must also be provided in the manuscript.

Flow Cytometry

Plots

Confirm that:

☒ The axis labels state the marker and fluorochrome used (e.g. CD4-FITC).

☒ The axis scales are clearly visible. Include numbers along axes only for bottom left plot of group (a 'group' is an analysis of identical markers).

☒ All plots are contour plots with outliers or pseudocolor plots.

☒ A numerical value for number of cells or percentage (with statistics) is provided.

Methodology

Sample preparation	Tumors were minced with scalpel, and digested with 1 mg/ml collagenase IV (Sigma, C2674-1G) and DNAse I at 1 mg/ml (Sigma, 11284932001) in DMEM supplemented with 1% FCS and 1% HEPES. Tumor draining lymph nodes (tdLN, inguinal) were mechanically grinded with glass slides.<br>Surface staining of mouse cells was performed in PBS, 1%BSA, 0,05% azide (Invitrogen) For intracellular staining cells were fixed and permeabilized using Fixation and Permeabilization Buffer kit (00-5523-00, eBiosciences) according to the manufacturer's protocol. For cytokine staining cells were incubated with brefeldin A (eBioscience), for four hours, For p-SMAD2/3 staining, cells were immediately fixed with Fixation and Permeabilization Buffer kit (00-5523-00 eBiosciences).<br>For cell sorting, T-cells were enriched with Pan T cell isolation kit II mouse (Miltenyi Biotec)
Instrument	Cells were stored on FACS ARIA II sort and analyzed on BD LSR Fortessa sort
Software	DIVA, 8.01 for BD LSR Fortessa sort<br>DIVA 8.03 for FACS ARIA II sort<br>Analysis with Floe Joe version X

Cell population abundance	After cell sorting 96-98% of purity was obtained
Gating strategy	After removing doublet, a gate was drawn on FCS/SSC to remove debris. Dead cells were excluded suing viability maker. Population of interest will be then analyzed by slecting the right axis in the "mother gate". Quadrant stat as well histogram limits were positionned based on FMO values

☒ Tick this box to confirm that a figure exemplifying the gating strategy is provided in the Supplementary Information.
